# Failure to respond to endogenous or exogenous melatonin may cause nonphotoresponsiveness in Harlan Sprague Dawley rats

**DOI:** 10.1186/1740-3391-3-12

**Published:** 2005-09-14

**Authors:** Matthew Rocco Price, Julie Anita Marie Kruse, M Eric Galvez, Annaka M Lorincz, Mauricio Avigdor, Paul D Heideman

**Affiliations:** 1 Department of Biology, College of William and Mary, Williamsburg, VA 23187, USA

## Abstract

**Background:**

Responsiveness to changing photoperiods from summer to winter seasons is an important but variable physiological trait in most temperate-zone mammals. Variation may be due to disorders of melatonin secretion or excretion, or to differences in physiological responses to similar patterns of melatonin secretion and excretion. One potential cause of nonphotoresponsiveness is a failure to secrete or metabolize melatonin in a pattern that reflects photoperiod length.

**Methods:**

This study was performed to test whether a strongly photoresponsive rat strain (F344) and strongly nonphotoresponsive rat strain (HSD) have similar circadian urinary excretion profiles of the major metabolite of melatonin, 6-sulfatoxymelatonin (aMT6s), in long-day (L:D 16:8) and short-day (L:D 8:16) photoperiods. The question of whether young male HSD rats would have reproductive responses to constant dark or to supplemental melatonin injections was also tested. Urinary 24-hour aMT6s profiles were measured under L:D 8:16 and L:D 16:8 in young male laboratory rats of a strain known to be reproductively responsive to the short-day photoperiod (F344) and another known to be nonresponsive (HSD).

**Results:**

Both strains exhibited nocturnal rises and diurnal falls in aMT6s excretion during both photoperiods, and the duration of the both strains' nocturnal rise was longer in short photoperiod treatments. In other experiments, young HSD rats failed to suppress reproduction or reduce body weight in response to either constant dark or twice-daily supplemental melatonin injections.

**Conclusion:**

The results suggest that HSD rats may be nonphotoresponsive because their reproductive system and regulatory system for body mass are unresponsive to melatonin.

## Introduction

Responsiveness of the reproductive system, metabolic rate, and other traits to changing photoperiods from summer to winter seasons is an important physiological trait in most temperate-zone mammals [1]. Seasonal changes in photoperiod, or day length, modify reproductive timing in many temperate-zone mammals including sheep, hamsters, rodents, horses, and ferrets by acting through the photoperiod pathway [2-4]. The photoperiod pathway transduces the photoperiod into a physiological signal beginning with the transduction of light or dark input from specialized photoreceptors and ganglion cells in the eye through the retinohypothalamic tract into two regions of the hypothalamus, the suprachiasmatic nucleus (SCN), and later the paraventricular nucleus. A sympathetic norepinephrine signal from the SCN then passes to the hindbrain, the superior cervical ganglion in the spinal cord, and eventually to the pineal gland, which releases the indoleamine hormone melatonin [5]. Pinealoctyes within the pineal gland convert tryptophan into 5-hydroxytryptamine (serotonin), acetylate serotonin into N-acetylserotonin (NAT), and finally methylate NAT with the enzyme hydroxyindole-O-methyltransferase to form melatonin (N-acetyl-5-methoxytryptamine) [5]. In the presence of light, inhibition of NAT enzyme activity reduces melatonin synthesis, and thus melatonin is secreted from pinealocytes primarily in darkness. The duration of elevated melatonin provides a physiological signal for photoperiods [6]. Melatonin binds to one or more receptor types, MT1 or MT2, initiating cellular responses that apparently produce the physiological effects of this hormone [7,8].

The photoperiod pathway is crucial for regulation of seasonal function in most temperate zone animals [1] However, there is genetic variation in photoresponsiveness within and among species of rodents [9]. It has been proposed that this variation is likely to be important in animal function and evolution [9,10]. With respect to humans, there is debate over the function of the photoperiod pathway [11,12]. Recent reviews suggest that genetic variation in the pathway may have functional and medical significance in humans [13,14]. Thus, identifying the physiological basis of and consequences from genetic variation in photoperiodic responses may be useful in understanding mammalian variation in this trait, with potential relevance to humans as well. A potential cause of nonphotoresponsiveness is a failure to secrete or metabolize melatonin in a pattern that reflects photoperiod length. Such variation occurs in humans, and the clinical significance of atypically elevated or depressed melatonin levels is widely recognized in human sleep disturbances and clinical conditions [reviewed in [14,15]]. Reduced amplitude and duration of nocturnally elevated melatonin is characteristic of a wide range of psychiatric disorders, including major depression and bipolar affective disorder [16].

Patterns of melatonin secretion can be estimated by the pattern of excretion of the primary metabolite of melatonin, 6-sulfatoxymelatonin (aMT6s) [17-19]. After synthesis, melatonin is rapidly metabolized in the liver and kidney by hydroxylation and subsequent sulfonation to produce aMT6s for later excretion in urine [18,19]. Because of the relatively rapid conversion of melatonin, it has been argued that melatonin secretion patterns are related to the amount of aMT6s present in urine, and aMT6s has been used as an indirect estimator of periods of elevated circulating melatonin [5]. However, this estimate can be imprecise because some melatonin is metabolized by other pathways, the conversion rate to aMT6s may vary genetically, and urine may be held in the bladder for some time before micturition.

Laboratory rats vary genetically in their responses to short-day photoperiods (eight hours light, 16 hours dark; SD). Some strains are functionally non-photoperiodic [2,20], including Sprague Dawley rats from Harlan USA (HSD) [21], though such strains are sometimes reproductively photoresponsive if a short photoperiod is combined with secondary cues such as food restriction, testosterone treatment, or olfactory bulbectomy [2,22]. In contrast, many other strains, including Fisher 344 (F344), Brown Norway (BN), ACI, BUF, and PVG inbred rat strains, are robustly reproductively photoresponsive, thereby demonstrating the presence of rat inter-strain variation in physiological and reproductive responses to short photoperiods [23-25]. Exposure to short photoperiods alone causes changes in F344 and BN reproductive organ size, food intake, and body weight [26]. Even stronger responses occur when food restriction or neonatal testosterone treatment is combined with short photoperiod treatment [21,24].

In the present study, tests were performed to find out whether the aMT6s urinary excretion pattern would vary between short and long photoperiods in young photoresponsive F344 and nonphotoresponsive HSD rats. We chose these two strains because young F344 rats have the greatest response to short photoperiod reported in rats, and HSD rats are the only strain for which there is clear evidence for a lack of response to short photoperiod [21,23,27]. In order to further examine the effects of photoperiod on melatonin, the question of whether young HSD rats would exhibit inhibition of reproductive development in response to constant dark or supplemental timed injections of melatonin was tested. As a photoperiodic strain, it was predicted that young F344 rats would have nocturnally elevated aMT6s, and that the duration of elevation would be longer in short photoperiods. Because non-manipulated young HSD rats are not photoperiodic [23], it was hypothesized that young HSD rats might lack nocturnally elevated aMT6s as an underlying cause of their nonphotoresponsiveness, or that any rise in aMT6s would not differ between long and short photoperiods in non-manipulated individuals. It was also hypothesized that if melatonin secretion was inadequate, low, or absent in young HSD rats, supplemental melatonin or constant dark might suppress reproductive development. An alternative hypothesis is that young HSD rats are normally nonresponsive not because of deficiencies in the pattern of nocturnally elevated melatonin, but because of a lack of response to short-day patterns of elevated melatonin. Under the alternative hypothesis, it was predicted that both strains would produce a nocturnal rise in aMT6s excretion and differences between long and short photoperiods in aMT6s excretion.

## Methods

### Experiment 1. aMT6s Excretion Patterns in F344 and HSD rats

This experiment used a 2 × 2 design with HSD and F344 rats in short-day (L8:D16; lights on at 0900; SD) and long-day (L16:D8; lights on at 0500; LD) photoperiods (n = 12 rats/treatment group). Breeder rats of the inbred Fischer F344 NHsd and outbred HSD strains from Harlan Sprague Dawley (Indianapolis, IN) were bred in polypropylene cages in LD photoperiod (40 × 23 × 23 cm) with stainless-steel wire tops and bedding of pine shavings. Harlan Teklad rodent diet (Indianapolis, IN) and tap water were provided ad libitum. Relative humidity was 40–65%, and temperature was maintained at 23 ± 3°C. Due to bright light's ability to cause retinal damage to albino rats, light intensity was maintained between 100 and 300 lux, as measured five cm above the cage floor. After weaning at age 21–24 days in LD, twelve young rats from each strain were transferred to SD, while twelve rats from each strain remained in LD. All were housed individually in polypropylene cages (33 × 20 × 20 cm). To avoid inconsistencies in aMT6s secretion due to the estrus cycles of female rats [28], only male rats were used in this study.

At age 7 to 8 weeks (± 3 days), when F344 rats are highly photoperiodic but HSD rats are not [23], rats were transferred to hanging cages (27 × 20 × 20 cm) with wire mesh bottoms and funnels to collect urine. Rats were given ad libitum tap water and fed a liquid diet reported to be complete for rats (Osmolite HN, Ross Laboratories, Columbus, OH) to stimulate urine secretion [17]. Lighting remained as above. Rats were then given 3 to 4 days to acclimate to cage and diet changes. At 15-minute sampling intervals over two consecutive 24-hour periods, urine was automatically collected (Eldex Universal Fraction Collector, Eldex Laboratories, Inc., Napa, CA). After each of the two 24-hour collection periods, each sample was weighed to determine urinary output volume, and samples were stored at -20°C. Concentration and volume changes due to evaporation over the collection period were corrected against a water evaporation control for each day of collection. Groups of eight successive 15-minute samples were combined to create two-hour sample periods, covering periods beginning at 0100, 0300, 0500, 0700, 0900, 1100, 1300, 1500, 1700, 1900, 2100, and 2300 hours. Finally, because pilot studies indicated that single 24-hour periods were missing urine samples from some two-hour collection periods from some animals, corresponding samples from the same time periods in the first and second days of collection were combined. The result produced urine samples from periods two hours in duration on successive nights from the same time period, with 12 such two-hour sample periods per individual. Urine samples were assayed for aMT6s with a 6-sulfatoxymelatonin ELISA kit (Buhlmann Laboratories, Allschwil, Switzerland) according to the manufacturer's protocol. Inter-assay coefficient of variation (CV) was 17% and intra-assay CV was 10% for standards near the midrange of values in this study. Data analysis treated each two-hour sampling interval as a single data point.

### Experiment 2. Effects of Constant Dark on HSD Rats

This experiment tested whether constant dark might provide a physiological signal that would suppress reproduction (as measured by gonad or seminal vesicle size) or body mass in HSD rats. HSD rats were raised until weaning at age 21 days in LD. At that time, one group of rats was transferred to SD (n = 13), and another group to constant dark (n = 11). After four weeks of treatment, rats were euthanized and body mass, paired testis mass, and paired seminal vesicle mass (emptied of fluid contents) were recorded.

### Experiment 3. Effects of Supplemental Melatonin on HSD Rats

This experiment tested whether supplemental melatonin might provide a physiological signal that would suppress reproduction (as measured by gonad or seminal vesicle size) or body mass in HSD rats. HSD rats were raised until weaning at age 21 days in LD. At that time, all rats were transferred to SD (lights on at 0900 h and lights out at 1700 h). For the following four weeks, one group (n = 24) was given S.C. injections of melatonin twice daily (100 μg of melatonin dissolved in 0.1 ml of 10% ethanol and 90% physiological saline), and a control group (n = 23) was injected with ethanolic saline vehicle. Injections were given twice daily at 1230 and 1500 hours. Single injections of this amount of melatonin at 1500 hours in SD suppressed reproduction and inhibited growth in F344 rats [27]. The injection at 1230 hours was included in this experiment because pilot data suggested a single injection did not affect young HSD rats. After four weeks of treatment, rats were euthanized and body mass, paired testis mass, and paired seminal vesicle mass (emptied of fluid contents) was recorded.

### Data Analysis

In statistical testing of data on aMT6s, the data from each strain was analyzed independently for nocturnally elevated aMT6s excretion and for differences in excretion between SD and LD. Variation in mean aMT6S was assessed with ANOVA (Statview 4.5), with photoperiod as the factor. Comparisons for equality of variance indicated no significant differences in variance between photoperiods or between strains. The researchers conducted a final set of analyses comparing the two strains, with both photoperiod and strain as factors, to test for clear differences between strains that might be related to photoresponsiveness. The strain comparison was considered statistically appropriate because this experiment was testing a prediction derived from other information that HSD rats would be different in an estimator of melatonin rhythms.

Unpaired t-tests were used to compare effects of constant dark or supplemental melatonin on body mass, testis mass, and seminal vesicle mass in experiments two and three.

All procedures were conducted in accordance with the *Guide for Care and Use of Laboratory Animals *and approved by the Research on Animal Subjects Committee (RASC) of the College of William and Mary.

## Results

### Experiment 1. aMT6s Excretion Patterns in F344 and HSD rats

**Figure 1:**
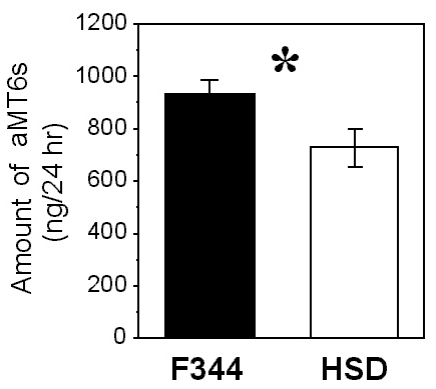
**Total urinary 6-sulfatoxymelatonin production in ng per 24 h for F344 and HSD rats**. Asterisk indicates P < 0.05. For each strain, n = 24 rats.

**Figure 2:**
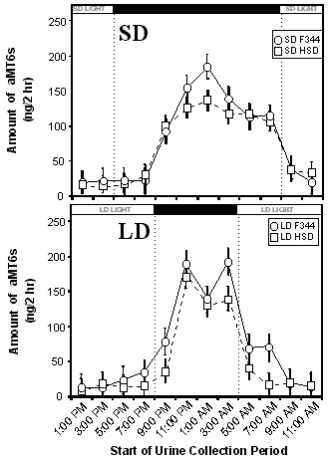
**24-hour urinary 6-sulfatoxymelatonin excretion rhythms (ng/2 h) for F344 and HSD rats in SD (upper panel) and LD (lower panel)**. Values shown are means +/- SEM. Bars at the top of the figure indicate periods of light and dark for the SD and LD treatments, respectively. For each treatment group, n = 12 rats.

F344 rats excreted significantly more total aMT6s than HSD rats (F = 4.22, P < 0.05, n = 24 for each strain; Fig. 1). Because F344 rats at these ages are 30% lighter in weight than HSD rats at the ages tested in this experiment [unpublished data and [23]], differences in excretion would be even more pronounced if expressed as excretion per unit body mass, with F344 rats excreting approximately 40% more aMT6s per unit body weight than HSD rats. Total aMT6s excretion did not differ significantly between SD and LD (F = 1.63, P = 0.21). There was a diurnal pattern of aMT6s excretion in both strains, with the lowest levels near the middle of the light period and the highest levels near the middle of the dark period (Fig. 2).

In SD, the pattern of excretion of aMT6s was very similar for the two strains of rats (Fig. 2). Excretion of aMT6s began rising in the collection period beginning at 9:00 pm, four hours after the onset of dark. Levels of aMT6s remained elevated, relative to the light period, through the remaining five collection periods of the dark period. The duration of excretion of aMT6s did not differ significantly between the two strains during the SD dark period (Repeated Measures ANOVA, F = 0.89, P = 0.35).

In LD, the pattern of excretion of aMT6s differed between the strains of rats (Fig. 2). Across the total dark period, there was an insignificant statistical trend (Repeated Measures ANOVA, F = 2.79, P = 0.098) for a higher level of excretion of aMT6s in F344 rats than in HSD rats. In the two collection periods immediately after the end of the dark period, aMT6s excretion was significantly higher in F344 rats than in HSD rats (Repeated Measures ANOVA, F = 12.22, P < 0.001).

In both strains of rats, aMT6s excretion was elevated for a longer duration in SD than in LD, but this difference between photoperiods was more pronounced in HSD rats (Fig. 2). In both strains, aMT6s excretion was significantly higher in SD than in LD in the 0500 and 0700 collection periods (F344: Repeated Measures ANOVA, F = 4.20, P < 0.05; HSD: Repeated Measures ANOVA, F = 11.64, P < 0.001). In the collection periods beginning at 5:00 pm or 7:00 pm for each strain, aMT6s excretion was low in both SD and LD (Fig. 2). Finally, unlike the case for F344 rats, HSD rats in the collection period beginning at 9:00 pm excreted lower levels of aMT6s in LD than in SD (Fig. 2; F = 5.02, P = 0.03).

**Figure 3:**
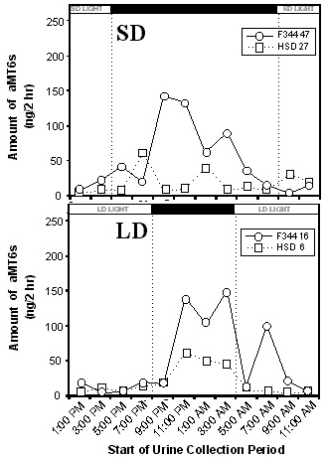
**24-hour urinary 6-sulfatoxymelatonin excretion rhythms (ng/2 h) for two individual F344 rats (one in SD and one in LD) and two individual HSD rats (one in SD and one in LD)**. Bars at the top of the figure indicate periods of light and dark for the SD (upper panel) and LD (lower panel) treatments. The four rats selected for presentation were those with the lowest total 6-sulfatoxymelatonin excretion in their respective treatment groups.

Some HSD rats either lacked a clear diurnal pattern of aMT6s excretion or had a very low amplitude nocturnal rise. In contrast, all F344 rats had a clear diurnal pattern of aMT6s with a robust nocturnal rise in aMT6s excretion. For example, the two F344 rats in SD and LD with the lowest total aMT6s excretion for their treatment groups nonetheless had a robust nocturnal rise in aMT6s excretion (Fig. 3). In contrast, the two HSD rats in SD and LD with the lowest total aMT6s excretion for their treatment groups had poorly developed rhythms of aMT6s excretion (Fig. 3).

### Experiment 2. Effects of Constant Dark on HSD Rats

**Figure 4:**
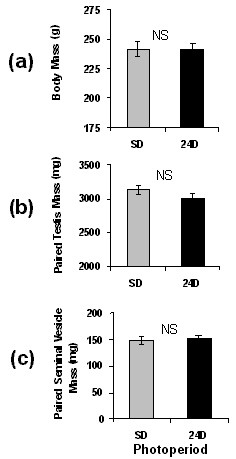
**Mean (+/- SEM) of body mass (a), paired testis mass (b), and paired seminal vesicle mass (c) of young HSD rats held in SD or constant dark (24D)**. Sample sizes: n = 13 in SD and 11 in 24D. NS indicates a lack of significant differences.

HSD rats held in constant darkness for four weeks following weaning did not differ from SD controls in body mass, testis mass, or seminal vesicle mass (Fig. 4, P > 0.10 for all).

### Experiment 3. Effects of Supplemental Melatonin on HSD Rats

**Figure 5:**
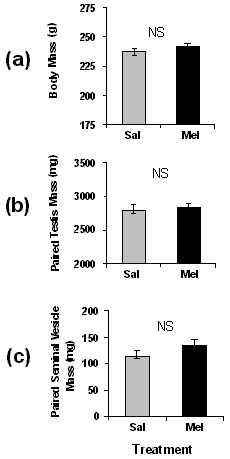
**Mean (+/- SEM) of body mass (a), paired testis mass (b), and paired seminal vesicle mass (c) of young HSD rats in SD treated with saline injections (Sal) or melatonin (Mel)**. Sample sizes: n = 23 in Sal and 24 in Mel. NS indicates a lack of significant differences.

HSD rats given twice daily injections of melatonin for four weeks did not differ from saline controls in body mass, testis mass, or seminal vesicle mass (Fig. 5, P > 0.10 for all).

## Discussion

Both strains of rats were found to have generally higher levels of excretion in the dark period than in the light period (Fig. 2), and both had a longer duration of nocturnally elevated aMT6s excretion in SD than in LD. These differences between SD and LD were as apparent in HSD rats as in F344 rats (Fig. 2). This suggests that, based on the pattern of aMT6s excretion, both HSD rats and F344 rats should be able to use melatonin secretion as a physiological signal to distinguish SD and LD. The data for young HSD rats on the nocturnal rise of aMT6s excretion and approximate amounts of aMT6s excreted per hour are consistent with nocturnal rises in L12:D12 reported by Usui and colleagues [29] on older Sprague Dawley rats from a different source (Clea Japan, Tokyo). However, in a few HSD rats in this study the pattern of aMT6s excretion lacked a clear nocturnal rise or had only a slight nocturnal rise (Fig. 3). In HSD rats, neither four weeks of constant darkness nor four weeks of supplemental melatonin affected body mass or suppressed reproduction (Figs. 4 and 5). In previous tests on young F344 rats at the same age, four weeks of short photoperiod treatment suppressed reproductive development and somatic growth. Relative to rats in LD, testis mass in SD was lower by about 50%, seminal vesicle mass in SD was lower by 80%, and body mass in SD was lower by 10–20% [21,23,30]. Pinealectomy blocked effects of SD [23], and four weeks of melatonin injections in LD caused reproductive suppression, reduced body mass, and also enhanced the suppressive effects of SD in short days [27].

These results suggest that there is a nocturnal rise in nocturnal melatonin in both young HSD and young F344 rats and a difference in both strains between SD and LD (Fig. 2), but only young HSD rats fail to respond to changes in photoperiod and to exogenous melatonin. While there were significant statistical differences between strains, the differences were small and may not reflect differences in serum melatonin levels. In contrast, there is previous evidence that in F344 rats, the normal endogenous melatonin signal does not produce a maximal response to short photoperiods. Exogenous melatonin delivered to young F344 rats in SD as S.C. injections before the dark period resulted in greater reproductive inhibition and lower body weight than SD alone [27]. In this study, the presence of nocturnal rises in excretion of aMT6s and differences between SD and LD patterns for both strains, along with evidence for a failure of HSD rats to respond to supplemental melatonin, is consistent with the alternative hypothesis, which says that differences in photoresponsiveness arise from inter-strain differences in physiological mechanisms responsible for processing the melatonin signal, rather than from inadequate melatonin secretion. In a previous comparison of young rats of these two strains [31], there was an up to 2.5-fold higher specific binding of iodomelatonin in the brains of young F344 rats than young HSD rats. Significant differences between HSD and F344 rats were found in the thalamic paraventricular nucleus and reunions nucleus, but not in some other brain areas, including the SCN. This suggests that the response to melatonin signals might be different in HSD and F344 rats, even if those melatonin signals were identical.

Young F344 rats excreted 25% more aMT6s than same-age HSD rats over two-day collection periods (Fig. 1), despite body weights that are approximately 30% lower at this age. This suggests that young HSD rats either secrete less melatonin than F344 rats or excrete a higher amount of melatonin and its metabolites through an alternative pathway (e.g., via the feces). The biological significance of this difference is not clear. However, it is possible that the small number of HSD rats that had little diurnal change in aMT6s (Fig. 3) may have too small a nocturnal rise in melatonin secretion for consistent responses to melatonin.

As in previous aMT6s studies in laboratory species [9,17,32-34] and human populations exposed to different photoperiods [35], substantial differences among individuals in amplitude and total excretion amount were observed within all four groups (e.g., Fig. 3). While some of this variation might be due to variation in urination pattern, the variation in total amount of aMT6s excreted should be only slightly affected by variation in urination of rats on a liquid diet. Due to the fact that inbred F344 rats are highly genetically similar, this suggests substantial environmental influences on melatonin secretion patterns, even in a highly controlled laboratory environment.

Variation in melatonin receptor number, density, or location have been implicated as potential sources of variation in this pathway in other species [31,36]. Differences in photoresponsiveness might also be attributable to variation in neurotransmitter systems mediating reproductive responses to melatonin, including negative feedback sensitivity to sex steroids or the influence of additional cues, such as food intake [9,27]. This is consistent with the suggestion that clinically significant circadian dysfunction in humans may occur downstream of melatonin production, or that both downstream as well as upstream processing dysfunction could occur concurrently with melatonin production dysfunction [37].

## Conclusion

Both strains of rats in both photoperiods exhibited nocturnal rises and diurnal falls in aMT6s excretion, and the duration of the nocturnal rise was longer in short photoperiod treatments in both. In addition, young HSD rats failed to suppress reproduction or reduce body weight in response to either constant darkness or twice-daily supplemental melatonin injections. In combination, these results suggest that HSD rats may be nonphotoresponsive because their reproductive system and the regulatory system for body mass are unresponsive to melatonin.

## Competing interests

The author(s) declare that they have no competing interests.

## Authors' contributions

**MEG **designed and conducted pilot experiments on sulfatoxymelatonin excretion and contributed to text on Experiment 1.

**MRP **and **JAMK **designed and conducted experiment 1; **MRP **carried out the assays and final analysis, and had the lead role in writing and revising the manuscript.

**AML **and **MA **designed and conducted Experiments 2 and 3, and **AML **conducted the data analyses and wrote text for Experiments 2 and 3.

**PDH **supervised the experiments and analysis, and finalized figures and text.
